# Differential brain *ADRA2A* and *ADRA2C* gene expression and epigenetic regulation in schizophrenia. Effect of antipsychotic drug treatment

**DOI:** 10.1038/s41398-021-01762-4

**Published:** 2021-12-20

**Authors:** Iria Brocos-Mosquera, Patricia Miranda-Azpiazu, Carolina Muguruza, Virginia Corzo-Monje, Benito Morentin, J. Javier Meana, Luis F. Callado, Guadalupe Rivero

**Affiliations:** 1 Department of Pharmacology, University of the Basque Country, UPV/EHU, Leioa, Bizkaia Spain; 2 Centro de Investigación Biomédica en Red de Salud Mental (CIBERSAM), Leioa, Spain; 3 Section of Forensic Pathology, Basque Institute of Legal Medicine, Bilbao, Spain; 4 Biocruces Bizkaia Health Research Institute, Barakaldo, Bizkaia Spain

**Keywords:** Schizophrenia, Epigenetics in the nervous system, Biomarkers

## Abstract

Postsynaptic α_2A_-adrenoceptor density is enhanced in the dorsolateral prefrontal cortex (DLPFC) of antipsychotic-treated schizophrenia subjects. This alteration might be due to transcriptional activation, and could be regulated by epigenetic mechanisms such as histone posttranslational modifications (PTMs). The aim of this study was to evaluate *ADRA2A* and *ADRA2C* gene expression (codifying for α_2_-adrenoceptor subtypes), and permissive and repressive histone PTMs at gene promoter regions in the DLPFC of subjects with schizophrenia and matched controls (*n* = 24 pairs). We studied the effect of antipsychotic (AP) treatment in AP-free (*n* = 12) and AP-treated (*n* = 12) subgroups of schizophrenia subjects and in rats acutely and chronically treated with typical and atypical antipsychotics. *ADRA2A* mRNA expression was selectively upregulated in AP-treated schizophrenia subjects (+93%) whereas *ADRA2C* mRNA expression was upregulated in all schizophrenia subjects (+53%) regardless of antipsychotic treatment. Acute and chronic clozapine treatment in rats did not alter brain cortex *Adra2a* mRNA expression but increased *Adra2c* mRNA expression. Both *ADRA2A* and *ADRA2C* promoter regions showed epigenetic modification by histone methylation and acetylation in human DLPFC. The upregulation of *ADRA2A* expression in AP-treated schizophrenia subjects might be related to observed bivalent chromatin at *ADRA2A* promoter region in schizophrenia (depicted by increased permissive H3K4me3 and repressive H3K27me3) and could be triggered by the enhanced H4K16ac at *ADRA2A* promoter. In conclusion, epigenetic predisposition differentially modulated *ADRA2A* and *ADRA2C* mRNA expression in DLPFC of schizophrenia subjects.

## Introduction

Schizophrenia is a chronic and disabling disease that affects around 1% of the world population [1]. Schizophrenia is recognized by positive (including delusions and hallucinations), negative (including lack of motivation and social withdrawal), and cognitive symptoms (including disturbances in working memory, selective attention, and learning). Noradrenergic system and its α_2_-adrenoceptors have been proposed to play a role in the pathophysiology and treatment of schizophrenia [2]. Among the three α_2_-adrenoceptor subtypes (α_2A_, α_2B_, and α_2C_), α_2A_- and α_2C_-adrenoceptors show the broadest distribution in central nervous system and they could be relevant in mental disorders as schizophrenia due to their specific role in memory and cognition [3]. Preclinical and clinical data have suggested that selective α_2A_-adrenoceptor agonists and α_2C_-adrenoceptor antagonists can ameliorate cognitive deficits [3–7]. The α_2_-adrenoceptor subtype-selective effects might be ascribed to their differential synaptic location in human dorsolateral prefrontal cortex (DLPFC) [8], a brain region that controls cognitive processes and emotions. In detail, presynaptically located α_2_-adrenoceptors might affect neuropsychiatric symptoms due to their effect on neurotransmitter feedback and regulation, whereas postsynaptic α_2_-adrenoceptors in DLPFC (mainly α_2A_-adrenoceptor subtype) may be critical in the regulation of cognitive functions as working memory [6]. In line with this, we recently reported postsynaptic α_2A_-adrenoceptor subtype upregulation in schizophrenia subjects [9]. This finding was specific for those schizophrenia subjects that were under antipsychotic treatment.

Schizophrenia’s etiology has been proposed to be multifactorial, because of the multiple small-effect but fewer large-effect susceptibility gene variants identified [10, 11]. α_2A_- and α_2C_-adrenoceptors are codified by *ADRA2A* and *ADRA2C* genes, which are located on chromosomes C10 and C4, respectively. Candidate gene studies have not found any association between polymorphisms at *ADRA2A/C* genes and schizophrenia [12–15]. Environmental factors may also contribute to the disorder’s etiology by their impact on epigenetic mechanisms [16, 17]. Epigenetics confers short- and long-term gene expression changes with no alteration of the DNA code and comprises mechanisms such as DNA methylation, histone post-translational modifications (PTMs), chromatin remodeling, and expression of noncoding RNAs. Several evidence suggests that histone PTMs contribute to the development of schizophrenia. Some of these findings are: altered expression of various enzymes, such as increased expression of histone methyltransferases (HMT) [18–20], histone deacetylase 1 (HDAC1) [21, 22], and reduced expression of HDAC2 [23, 24] in prefrontal cortex (PFC) of schizophrenia subjects. Studies of chromatin immunoprecipitation followed by sequencing (ChIP-Seq) in human brain have found an overrepresentation of open chromatin-associated modifications [histone H3 lysine 4 trimethylation (H3K4me3 and histone H3 lysine 27 acetylation (H3K27ac)] in schizophrenia-related genes, this finding being specific for neuronal cells [25]. Actually, studies in brain cortex of schizophrenia subjects have identified altered histone H3 acetylation and methylation at promoter regions of certain genes [26–28]. More recently, ChIP-seq evaluation of cell-type H3K4me3 in PFC of schizophrenia subjects has revealed individual alterations in neurons [29]. These findings, along with the observed HDAC inhibitory activity of mood stabilizer valproate when administered at therapeutic doses in schizophrenia [30, 31] suggest the importance of histone PTMs in schizophrenia.

The present study aimed to assess the mRNA expression of *ADRA2A* and *ADRA2C* in postmortem DLPFC of schizophrenia subjects. Due to the relevance of epigenetic changes in schizophrenia and given the possibility that histone PTMs at promoter regions could regulate *ADRA2A* and *ADRA2C* mRNA expression, we also evaluated PTMs of histone H3 (H3K4me3, H3K27me3, H3ac, H3K9ac, H3K27ac) and H4 (H4K5ac and H4K16ac) at promoter regions of both *ADRA2A* and *ADRA2C* genes. In addition, we evaluated the possible modulation of mRNA expression and epigenetic mechanisms by antipsychotic treatment by comparing schizophrenia subjects according to antipsychotic drug presence or absence in blood at the time of death.

## Materials and methods

### Postmortem human brain samples

Schizophrenia and control subjects included in this study were the same 48 subjects assessed in a previous work (see Brocos-Mosquera et al. [9] for detailed description). Briefly, human brain samples of DLPFC (Brodmann’s area 9, BA 9) were obtained at autopsies in the Basque Institute of Legal Medicine, Bilbao, Spain, and immediately stored at −70 °C until assay. As previously described [9], toxicological screening for the detection of antidepressants, antipsychotics, psychotropic drugs, and ethanol on blood was performed at the National Institute of Toxicology, Madrid, Spain.

Twenty-four subjects had an antemortem diagnosis of schizophrenia and were matched to 24 control subjects for sex, age, postmortem delay (time interval between death and autopsy, PMD), and storage time of the samples (ST, Table 1). Schizophrenia subjects were divided in 12 antipsychotic-free (AP-free) and 12 antipsychotic-treated (AP-treated) subjects according to the presence or absence of antipsychotics in blood at the time of death. Out of the 24 matched pairs of schizophrenia and control subjects, 5 pairs had to be excluded from the *ADRA2A* and *ADRA2C* mRNA expression study due to technical reasons [RNA integrity number (RIN) value below 5 and/or absence of cDNA amplification for more than one gene]. Full description of demographic and toxicological characteristics of all subjects in mRNA and epigenetics studies is shown in respective Supplementary Tables 1 and 2. Seventeen out of the 24 schizophrenia subjects had died by suicide, with a similar distribution in AP-free and -treated subgroups (see Supplementary Table 3 for summarized demographic characteristics). A cohort of suicide victims with antemortem diagnosis of major depression and matched controls was used for hypothesis validation (*n* = 13, see Supplementary Table 4 for demographic characteristics). Samples from schizophrenia or major depression subjects and matched controls were always processed in parallel. The study was developed in accordance with legal policy and ethical review boards for postmortem brain studies.

**Table 1:** Sex, age, PMD, and ST of schizophrenia and control subjects included in the study.

	ALL		AP-free		AP-treated	
	24 C	24 S	12 C	12 S	12 C	12 S
Sex (M/F)	16/8	16/8	9/3	9/3	7/5	7/5
Age (years)	43 ± 2	43 ± 2	44 ± 2	43 ± 2	42 ± 3	42 ± 3
PMD (hours)	18 ± 2	16 ± 2	15 ± 3	17 ± 4	21 ± 3	15 ± 2
ST (months)	102 ± 12	98 ± 6	112 ± 21	96 ± 8	92 ± 11	100 ± 10

M, male, F, female; PMD, postmortem delay; ST, storage time of the samples; C, control subjects; S, schizophrenia subjects; AP-free, antipsychotic-free, AP-treated, antipsychotic-treated.

Quantitative variables are expressed as mean ± SEM (standard error of the mean).

### Animals and treatments

Animal housing and treatment were performed as already described [32]. Mice were randomly assigned to the different experimental groups. Briefly, male Sprague-Dawley rats were submitted to acute and chronic treatment with selected representative antipsychotic drugs with atypical and typical profile. Acutely treated rats were injected i.p. with saline (1 ml/kg, *n* = 5), risperidone (1 mg/kg, *n* = 5), clozapine (10 mg/kg, *n* = 4; both from Tocris, Bristol, UK) or haloperidol (1 mg/kg, *n* = 4; Sigma Aldrich, MO, USA) and sacrificed 3 h after the injection. Chronically treated rats were injected i.p., every 12 h, during 21 days, with saline (1 ml/kg, *n* = 6), risperidone (0.5 mg/kg, *n* = 6), clozapine (5 mg/kg, *n* = 6) or haloperidol (0.5 mg/kg, *n* = 6). Animals were sacrificed 48 h after the last injection of risperidone or clozapine and 72 h after that of haloperidol or saline. Brain cortex was dissected and stored at −70 °C until assays.

### mRNA expression and cDNA synthesis

Total RNA was extracted using commercial RiboPure^TM^ kit (Thermo Fisher Scientific, Waltham, MA, USA) according to the manufacturer’s instructions. RNA concentration and quality were measured in a NanoDrop 1000 Spectrophotometer (Thermo Fisher Scientific). RIN was also assessed for RNA quality in the Agilent 2100 Bioanalyzer (Agilent, Santa Clara, CA, USA) using Agilent RNA 6000 Nano kit and RNA Nano chips following the manufacturer’s instructions (see Supplementary Tables 1 and 4). Extracted human RNA underwent DNase digestion using the Deoxyribonuclease I, Amplification Grade (Thermo Fisher). 1 µg of total RNA was converted to single-stranded cDNA using High-Capacity cDNA Reverse Transcription kit (Thermo Fisher) following the manufacturer’s instructions.

### Chromatin immunoprecipitation (ChIP) assay

Human postmortem brain (120 mg) was homogenized in 810 μl of douncing buffer (4 mM MgCl_2_, 1 mM CaCl_2_, and 10 mM Tris-HCl; pH = 7.5) and incubated with 1 U/ml of micrococcal nuclease (MNase; Sigma-Aldrich, St. Louis, MO, USA) for 10 min at 37 °C. The MNase activity was stopped by adding 10 mM EDTA. The sample was diluted by adding nine-fold volume excess of hypotonic lysis buffer [0.1 mM benzamidine, 0.1 mM phenylmethylsulfonylfluoride (PMSF), 1.5 mM 1,4-dithio-dl-threitol (DTT), and 0.2 mM EDTA; pH = 8.0] and then incubated on ice for 1 h. The sample was centrifuged at 1000 × *g* (5810 R centrifuge; Eppendorf, Hamburg, Germany) for 10 min at 4 °C to remove insoluble material. Soluble chromatin was diluted adding ten times concentrated incubation buffer (50 mM EDTA, 500 mM NaCl, and 200 mM Tris-HCl; pH = 7.5). 60 µl of diluted chromatin (input sample) were removed and saved at 4 °C until elution. Primary antibodies at different concentrations (see Supplementary Table 5) and 20 μl of fully suspended protein A magnetic beads (Millipore, Burlington, MA, USA) were added to aliquots of diluted chromatin and incubated overnight at 4 °C with rotation. Protein A magnetic beads were pelleted with the magnetic separator and the supernatant removed completely. Then, the protein A bead-antibody/chromatin complexes were washed by resuspending the pelleted beads in 0.5 ml each of the following cold buffers in this order: low salt (150 mM NaCl, 20 mM Tris-HCl, 2 mM EDTA, 0.1% SDS, 1% Triton X-100; pH = 8.1); high salt (500 mM NaCl, 20 mM Tris-HCl, 2 mM EDTA, 0.1% SDS,1% Triton X-100; pH = 8.1); LiCl immune complex wash buffer (250 mM LiCl, 10 mM Tris-HCl, 1 mM EDTA, 0.01% IGEPAL CA630, 0.01% deoxycholic acid; pH = 8.1) and TE buffer (1 mM EDTA, 10 mM Tris-HCl; pH = 8.1). Once resuspended in each washing buffer, beads were incubated for 20 min at 4 °C with rotation, followed by magnetic clearance and careful removal of supernatant fraction prior to the addition of next washing buffer. Elution of protein/DNA complexes to free DNA was carried out by incubation of immune complexes and input samples in ChIP elution buffer (1% SDS, 0.1 M NaHCO_3_) with proteinase K (100 ug/ml) at 62 °C for 2 h with shaking followed by an incubation at 95 °C for 10 min. Magnetic beads were separated from the complex antibody/chromatin and DNA in the supernatant of all samples (input included) was purified with QIAquick PCR purification kit (Qiagen, Hilden, Germany) following the manufacturer’s instructions. Final DNA volume was 1:4 diluted in molecular grade water.

### Quantitative real-time PCR

Quantitative real time PCR (qPCR) was performed on cDNA synthesized from isolated mRNA and on genomic DNA obtained from ChIP assays. qPCR reactions were carried out using Power or Fast SYBR Green Master Mix (Thermo Fisher, Waltham, MA) on a StepOne^TM^ system (Thermo Fisher) following the manufacturer’s instructions. The final volume for each reaction was 10 μl with either 20 ng of cDNA or 4 µl of genomic DNA (obtained from ChIP experiments) and corresponding gene specific primers (see Supplementary Table 6 for detailed specifications). Dissociation curve analyses were carried out at the end of each run for PCR product verification. mRNA expression of reference genes was assessed by pre-designed TaqMan^®^ assays (Supplementary Table 7). All samples were run in triplicates. Each run included a negative water control. See Supplementary Figs. 1–3 for detailed characterization of qPCR assays.

### Data analysis and statistical procedures

The mRNA expression of target genes *ADRA2A* and *ADRA2C* was corrected with that of reference genes *GAPDH* and *RPS13*, and with a reference sample (pool of control samples) using ΔΔCt method: ΔΔCt = (Ct (target gene)_sample_ – Ct (reference gene)_sample_) – (Ct (target gene)_reference sample_ – Ct (reference gene) _reference sample_). The relative amount of mRNA was calculated as 2^−ΔΔCt^. In the case of animal data, mRNA expression of *Adra2a* and *Adra2c* genes was corrected with that of *Gapdh* and *Rps29*. For ChIP data, fold changes relative to 6% of input DNA were determined using the comparative Ct method, where ΔCt = Ct (target gene)_immunoprecipitated DNA sample_ – Ct (target gene)_6% input DNA_. The relative amount of immunoprecipitated DNA was calculated as 2^−ΔCt^. Relative amounts of both mRNA and DNA were subjected to Grubbs’ test to detect any possible outlier.

Data analysis was performed with GraphPad Prism 8^©^ (GraphPad Software, Inc., San Diego, CA, USA), SPSS 26.0 (Chicago, IL, USA), and InVivoStat [33] programs, and results expressed as means ± SEM. Correlations between sex, age, PMD, ST, RIN, and experimental data were determined using multiple regression analysis. When significant, the relationship between numerical variables and experimental data was further studied by simple linear regression analysis. Statistical comparison of the means between control and schizophrenia subjects, and between males and females was performed by two-tailed Student’s t-test. If significant, statistical differences between means were studied by ANCOVA analysis with relevant variable as covariate. Statistical analysis of animal data was performed by one-way ANOVA test followed by Tukey’s multiple comparisons test. *P* values < 0.05 were considered statistically significant.

## Results

### Demographic and methodological variables contributing to *ADRA2A* and *ADRA2C* mRNA expression in human DLPFC

First, multiple regression analysis of data from control subjects was performed in order to study the contribution of nominal predictor variables (sex) and continuous predictor variables (age, PMD, ST, and RIN) to corrected *ADRA2A* and *ADRA2C* mRNA expression. A significant model emerged [F(1,17) = 9.568, *p* = 0.007]. The analysis revealed that corrected *ADRA2A* mRNA expression was significantly influenced by RIN of the samples (β = −0.6, *p* = 0.007), regardless of sex and age of the subjects and PMD and ST. Linear regression analysis showed a negative correlation between RIN and corrected *ADRA2A* mRNA expression (Supplementary Fig. 4). By contrast, corrected *ADRA2C* mRNA expression was not influenced by any of the variables. Therefore, when significant differences in *ADRA2A* mRNA expression between groups were observed, subsequent ANCOVA analyses with RIN as covariate were performed.

### *ADRA2A* and *ADRA2C* mRNA expression in postmortem human DLPFC of schizophrenia subjects and matched controls

**Fig. 1:**
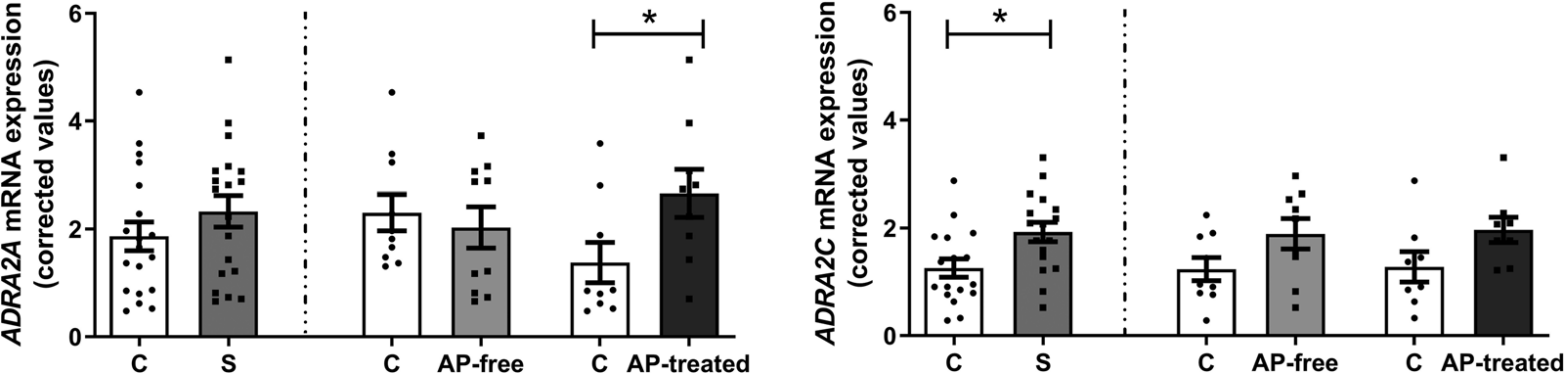
*ADRA2A* and *ADRA2C* mRNA expression in schizophrenia. Corrected *ADRA2A* and *ADRA2C* mRNA expression in DLPFC of schizophrenia subjects (S, *n* = 19), antipsychotic-free (AP-free, *n* = 10) and antipsychotic-treated (AP-treated, *n* = 9) subjects, and their respective matched controls (C). Data are shown as mean ± SEM. Statistical significance is denoted by *p < 0.05, unpaired two-tailed Student’s t-test.

*ADRA2A* mRNA expression in schizophrenia subjects was not statistically different from that in matched controls (Δ = +25%, *n* = 19, *p* = 0.25, Fig. 1A). Division of schizophrenia group by presence or absence of blood antipsychotics revealed a significant increase in *ADRA2A* mRNA expression in AP-treated subjects (Δ = +93% vs matched controls, *n* = 9, *p* = 0.042, Fig. 1A). As previously mentioned, RIN of the samples negatively influenced *ADRA2A* mRNA expression. Although RIN of the samples was not significantly different between AP-treated schizophrenia subjects and matched controls (see Supplementary Table 1), ANCOVA analysis with RIN as covariate was performed. ANCOVA analysis estimated that *ADRA2A* mRNA expression in AP-treated schizophrenia subjects was 94% higher than in matched controls but the difference did not reach statistical significance (*p* = 0.065).

In schizophrenia subjects *ADRA2C* mRNA expression was significantly higher than in matched controls (Δ = +53%, *n* = 17, *p* = 0.016, Fig. 1B). Analysis of schizophrenia subjects according to the presence or absence of antipsychotic treatment, revealed that *ADRA2C* mRNA expression was non-significantly enhanced over that in controls in both AP-free (Δ = +53%, *n* = 9, *p* = 0.136, Fig. 1B) and AP-treated schizophrenia subjects (Δ = +54%, *n* = 8, *p* = 0.088, Fig. 1B).

*ADRA2A* and *ADRA2C* mRNA expression in schizophrenia subjects could also be influenced by the fact that most schizophrenia subjects in the study were suicide victims (13 out of 19). In order to evaluate this possibility, we analyzed *ADRA2A* and *ADRA2C* mRNA expression in suicide victims with an antemortem diagnosis of major depression. Compared to matched controls, neither *ADRA2A* nor *ADRA2C* mRNA expression was significantly altered (Supplementary Fig. 5).

### Effect of antipsychotic treatment on *Adra2a* and *Adra2c* mRNA expression in rat brain cortex

**Fig. 2:**
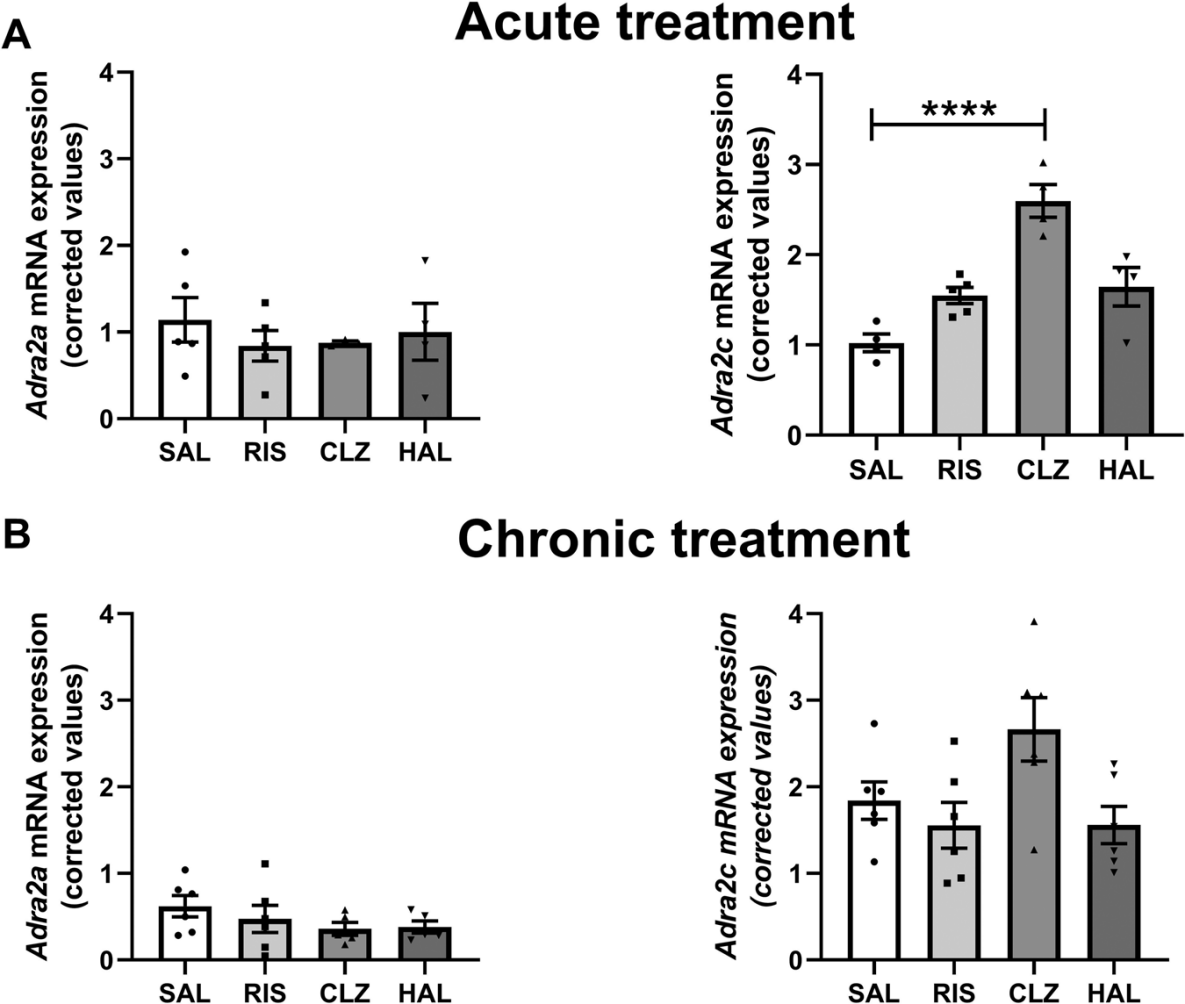
Effect of antipsychotic treatment on *Adra2a* and *Adra2c* mRNA expression in rat brain cortex. Corrected *Adra2a* and *Adra2c* mRNA expression in brain cortex of rats treated acutely (**A**) and chronically (**B**) with saline (SAL), risperidone (RIS), clozapine (CLZ), and haloperidol (HAL). Data are shown as mean ± SEM of 3−6 animals. Statistical analysis was performed by one-way ANOVA test followed by Tukey’s multiple comparison test. Statistical significance is denoted by ****p < 0.0001.

Considering the specific *ADRA2A* mRNA increase observed in AP-treated schizophrenia subjects, we aimed to evaluate the effect of acute and chronic antipsychotic treatment in rat brain cortex *Adra2a* and *Adra2c* mRNA expression. As observed in Fig. 2, *Adra2a* mRNA expression was modulated by neither acute nor chronic treatment with atypical (risperidone and clozapine) or typical antipsychotics (haloperidol). However, compared to saline-treated rats *Adra2c* mRNA expression was significantly increased in acute (Δ = +154% vs saline, *p* < 0.0001, Fig. 2A) and non-significantly increased in chronically clozapine-treated rats (Δ = +45% vs saline, *p* = 0.177, Fig. 2B).

### Demographic and methodological variables contributing to histone PTMs at *ADRA2A* and *ADRA2C* promoters in human DLPFC

The effect of demographic and methodological variables on histone PTMs at *ADRA2A* and *ADRA2C* promoters was individually analyzed. Significant models emerged for *ADRA2A* promoter-associated H3K27me3 [F(1,17) = 5.874, *p* = 0.027], H3ac [F(1,17) = 5.21, *p* = 0.036] and H4K5ac [F(1,17) = 5.194, *p* = 0.036]. H3K27me3 and H3ac at *ADRA2A* promoter were significantly influenced by sex of the subjects (β = 0.507, *p* = 0.027 and β = 0.484, *p* = 0.036, respectively), being higher in females than in males (Δ = +113% and Δ = +170%, respectively). H4K5ac at *ADRA2A* promoter gene was positively influenced by ST (β = 0.484, *p* = 0.036).

In the case of histone PTMs at *ADRA2C* promoter, significant models emerged for H3K27me3 [F(1,17) = 8.846, *p* = 0.009], H3K27ac [F(1,17) = 5.318, *p* = 0.034] and H4K16ac [F(1,17) = 5.561, *p* = 0.031]. H3K27me3 at *ADRA2C* promoter gene was influenced by sex of the subjects being higher in females than in males (β = 0.585, *p* = 0.009, Δ = +138%). At *ADRA2C* promoter H3K27ac was significantly influenced by PMD of samples (β=0.488, *p* = 0.034) whereas H4K16ac was influenced by ST (β = 0.496, *p* = 0.031). The effect of variables significantly contributing to experimental data is depicted in Supplementary Fig. 4.

### Histone PTMs at *ADRA2A* and *ADRA2C* promoters in postmortem human DLPFC of schizophrenia subjects

To evaluate the possible epigenetic modulation of altered *ADRA2A* and *ADRA2C* mRNA expression in schizophrenia subjects, histone modification signature comprised of histone methylations—permissive H3K4me3 and repressive H3K27me3—and histone acetylations—H3ac, H3K9ac, H3K27ac, H4K5ac, and H4K16ac—was evaluated at both *ADRA2A* and *ADRA2C* promoter regions.

**Fig. 3:**
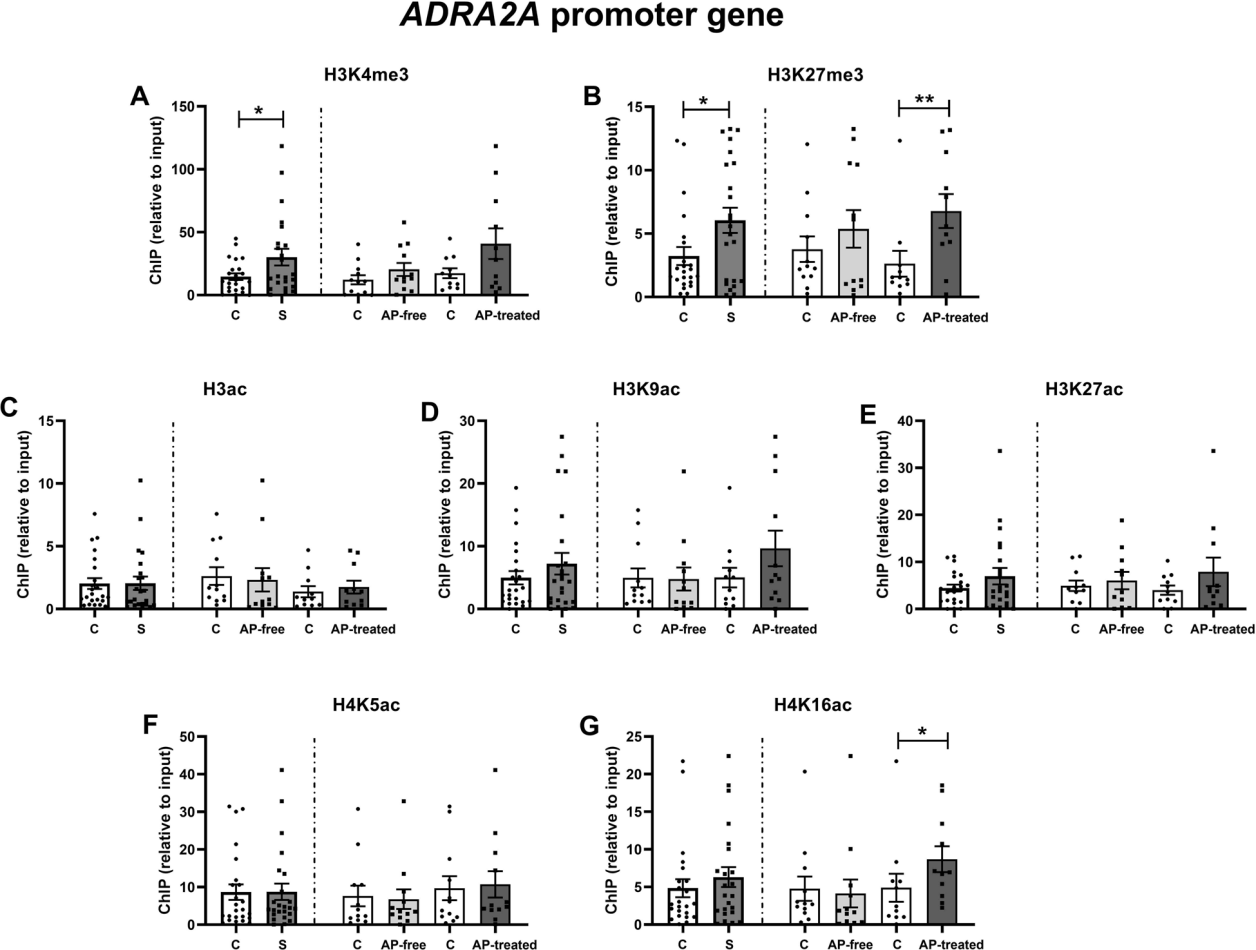
Histone PTMs at *ADRA2A* promoter in schizophrenia. Histone 3 methylation [H3K4me3 (**A**) and H3K27me3 (**B**)] and acetylation [H3ac (**C**), H3K9ac (**D**), H3K27ac (**E**)] and histone 4 acetylation [H4K5ac (**F**) and H4K16ac (**G**)] at *ADRA2A* promoter region. Data are shown as mean ± SEM. Statistical significance is denoted by *p < 0.05, **p < 0.01, paired two-tailed Student’s t-test. C control subjects, S schizophrenia subjects, AP-free antipsychotic-free, AP-treated antipsychotic-treated.

For *ADRA2A* promoter region, in schizophrenia subjects, both permissive H3K4me3 (Δ=+105%, *p* = 0.021, Fig. 3A) and repressive H3K27me3 (Δ = +86%, *p* = 0.01, Fig. 3B) were increased compared to matched controls. Division of schizophrenia subjects by presence or absence of blood antipsychotics, revealed that H3K27me3 at *ADRA2A* promoter was significantly increased in AP-treated schizophrenia subjects (Δ = +159% vs matched controls, *p* = 0.006, Fig. 3B). Although non-significantly, H3K4me3 at *ADRA2A* promoter was also increased in AP-treated schizophrenia subjects (Δ = +135% vs matched controls, *p* = 0.072, Fig. 3A). Separate analysis of AP-free and AP-treated schizophrenia subjects also revealed the selective increase of H4K16ac in AP-treated schizophrenia subjects (*D* = +82% increase, *p* = 0.029, Fig. 3G). The rest of the studied histone PTMs were not significantly different between the compared groups.

**Fig. 4:**
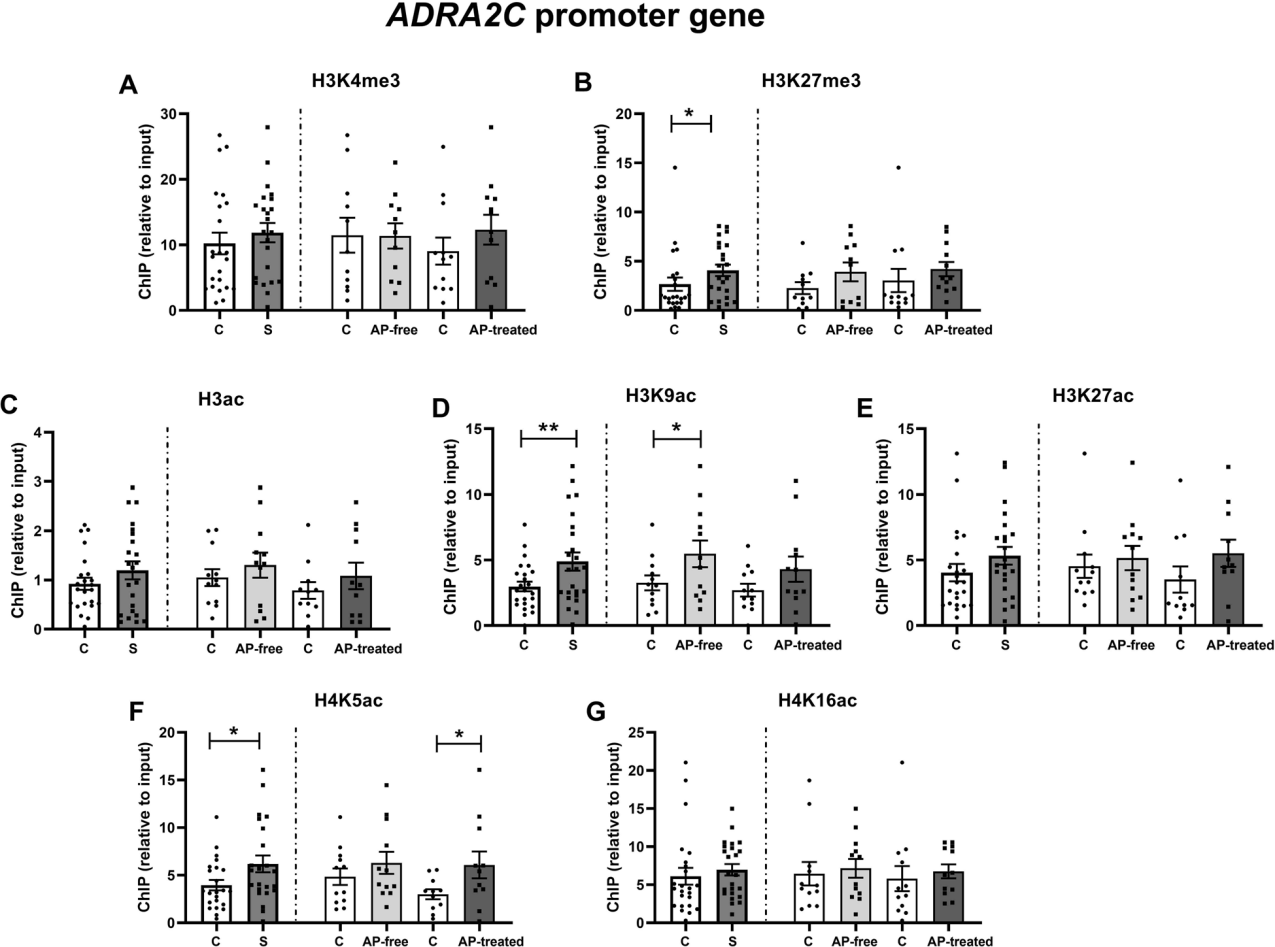
Histone PTMs at *ADRA2C* promoter in schizophreni. Histone 3 methylation [H3K4me3 (**A**) and H3K27me3 (**B**)] and acetylation [H3ac (**C**), H3K9ac (**D**), H3K27ac (**E**)] and histone 4 acetylation [H4K5ac (**F**) and H4K16ac (**G**)] at *ADRA2C* promoter region. Data are shown as mean ± SEM. Statistical significance is denoted by *p < 0.05, **p < 0.01, paired two-tailed Student’s t-test. C control subjects, S schizophrenia subjects, AP-free antipsychotic-free, AP-treated antipsychotic-treated.

The study of histone PTMs at *ADRA2C* promoter region in schizophrenia subjects showed a significant increase of H3K27me3 (Δ = +52%, *p* = 0.046, Fig. 4B), H3K9ac (Δ = +63%, *p* = 0.007, Fig. 4D) and H4K5ac (Δ = +55%, *p* = 0.027, Fig. 4F). Separate analysis of AP-free and AP-treated subjects showed that compared to matched controls, H3K9ac was increased in AP-free subjects (Δ = +67%, *p* = 0.043, Fig. 4D), whereas H4K5ac was increased in AP-treated subjects (Δ = +103%, *p* = 0.048, Fig. 4F). The rest of the studied histone PTMs were not significantly different between the compared groups.

In addition, none of the studied histone PTMs was found to be different between schizophrenia and control subjects at promoter region of reference gene *GAPDH* (Supplementary Fig. 6).

## Discussion

In this study, we observed a differential *ADRA2A* and *ADRA2C* mRNA expression in DLPFC of schizophrenia subjects. *ADRA2A* mRNA expression was selectively upregulated in AP-treated subjects, whereas *ADRA2C* mRNA expression was enhanced in schizophrenia subjects regardless of the presence or absence of antipsychotics in blood at the time of death.

The selective upregulation of *ADRA2A* mRNA expression in those schizophrenia subjects that showed detectable antipsychotic blood levels is in line with previous reports indicating a higher α_2A_-adrenoceptor protein expression in synaptosomal and postsynaptic membranes of the same AP-treated schizophrenia subjects [9]. In human PFC, α_2A_-adrenoceptor represents the main α_2_-adrenoceptor subtype [34–36]. Electron microscopy studies have shown α_2A_-adrenoceptors to be prevalent in neurons, showing presynaptic and postsynaptic location [37]. Subcellular fractionation of human DLPFC followed by immunolabeling showed the predominant postsynaptic location of α_2A_-adrenoceptors (95%) while only the remaining 5% is in the presynapse [8]. In this sense, due to mRNA predominant somatic location, and although certain *ADRA2A* mRNA expression in glial cells cannot be ruled out [37–39], we believe that most of the mRNA expression detected in our study may belong to postsynaptic neuronal mRNA. Moreover, present observations suggest that *ADRA2A* mRNA upregulation in AP-treated schizophrenia subjects might be the driving force in the upregulated postsynaptic α_2A_-adrenoceptor protein expression observed in the same subjects [9]. Postsynaptic neuronal expression of α_2_-adrenoceptors has been demonstrated on GABAergic interneurons [40] and on glutamatergic pyramidal neurons [41] of rat and rhesus monkey frontal cortex. Yet, the relevance of the enhanced mRNA and protein expression of α_2A_-adrenoceptors in AP-treated schizophrenia subjects is unknown. In DLPFC, postsynaptically located α_2A_-adrenoceptors play a relevant role in cognitive processes. From its postsynaptic location, α_2A_-adrenoceptors control noradrenergic tone on layer III pyramidal cells and upon stimulation by selective agonists, α_2A_-adrenoceptors enhance DLPFC-dependent working memory [42, 43]. Whether enhanced α_2A_-adrenoceptors in DLPFC of AP-treated schizophrenia subjects contributed to improve cognitive outcomes in these subjects is unknown.

By contrast, *ADRA2C* mRNA expression was upregulated in postmortem brain of schizophrenia subjects regardless of the presence of antipsychotics in blood. Evaluation of α_2C_-adrenoceptor protein expression in the same subjects revealed that α_2C_-adrenoceptor density in synaptosomal subcellular fractions was unaltered regardless of antipsychotic drug presence [9]. However, mRNA and protein expression do not necessarily need to be regulated in parallel [44] and the increase in *ADRA2C* mRNA expression in schizophrenia deserves further investigation. These results may suggest that *ADRA2C* upregulation would be a feature of schizophrenia pathology as opposed to α_2A_-adrenoceptor mRNA and protein expression selective upregulation in AP-treated schizophrenia subjects but other factors should also be considered. In most of the AP-treated schizophrenia subjects of this study, blood toxicology detected the presence of atypical antipsychotic drugs (Supplementary Table 1). In this sense, atypical antipsychotic drugs such as clozapine and risperidone show α_2_-adrenoceptor antagonistic properties with a preference for α_2C_- over α_2A_-adrenoceptor [5, 45, 46]. In order to study if α_2_-adrenoceptor antagonism by antipsychotic drugs could induce *ADRA2A* and *ADRA2C* mRNA expression, we conducted experiments in acutely and chronically AP-treated male rat brain cortex. Our results showed that *Adra2c* mRNA expression was upregulated in rats treated with either acute or chronic clozapine whereas *Adra2a* mRNA expression was unaltered. Although inclusion of female rats might have improved data validity, obtained results may be interpreted by pharmacological mechanisms. The selective upregulation of *Adra2c* mRNA expression triggered by acute and chronic clozapine but not by risperidone might relate to α_2_-adrenoceptor occupancy. In rodents, systemic administration of 5 mg/kg of clozapine occupies 65% of α_2A_- and 95% of α_2C_-adrenoceptors in brain [47], whereas 0.5 mg/kg risperidone only occupies 12% of α_2A_- and 19% of α_2C_-adrenoceptors [48]. Thus, increased *ADRA2C* mRNA expression in schizophrenia might be related to α_2C_-adrenoceptor antagonism by some atypical antipsychotic drugs. However, *ADRA2C* mRNA expression was non-significantly enhanced over control values in both AP-free and AP-treated schizophrenia subjects. It is worth mentioning that toxicological analysis in blood samples of AP-free schizophrenia subjects indicates that antipsychotics had not been present in an antemortem period of days but it does not discard previous antipsychotic usage by these subjects. Thus, modulation of *ADRA2C* mRNA expression by past antipsychotic drug usage in AP-free subjects might also be considered. On the other hand, the results in both acute and chronically-treated rats failed to explain the upregulation of *ADRA2A* mRNA expression in AP-treated schizophrenia subjects. Whether this lack of consistency is due to differences among species or due to a specific antipsychotic-induced modulation of *ADRA2A* mRNA expression in brains of schizophrenia subjects is unknown. An alternative approach would have been to study *ADRA2A* and *ADRA2C* mRNA expression in human brain of subjects treated with antipsychotic drugs but without schizophrenia diagnose. However, the limited availability of that kind of samples would have hindered the study. Thus, the hypothesis that antipsychotic drug effects on schizophrenia brains might differ from their effect on non-pathological brains points to the need of relevant animal models of schizophrenia. Actually, antipsychotic drugs have already shown differential effects on schizophrenia animal models compared to control animals [49].

As previously mentioned, another important aspect is that 13 of 19 schizophrenia subjects in the mRNA study died by suicide, with a similar distribution in AP-free and AP-treated subgroups. α_2_-adrenoceptors have been reported to be increased in postmortem brain of suicide victims [50] with vast literature reporting upregulated α_2_-adrenoceptors in brains of suicide victims with an antemortem diagnosis of major depression (see [51–54] and references within). However, information on α_2A_ and α_2C_-adrenoceptor subtype delineation is still lacking. Thus, we aimed to evaluate if enhanced *ADRA2C* mRNA expression in these schizophrenia subjects could be related to suicide completion by using a cohort of 13 depressed suicide victims. The results showed that neither *ADRA2A* nor *ADRA2C* mRNA were altered in depressed suicide victims as opposed to results in the schizophrenia cohort. Therefore, modulation of *ADRA2C* mRNA expression due to suicidal completion does not seem plausible.

Epigenetics may be particularly relevant for understanding schizophrenia [17]. Besides literature on DNA methylation studies in schizophrenia and the growing number of studies on miRNA [55], several findings indicate that histone PTMs may play a role in the etiology and pathophysiology of schizophrenia [26–29]. Thus, the association of several histone H3 and histone H4 PTMs at *ADRA2A* and *ADRA2C* promoter regions was studied. The results show specific histone PTMs at *ADRA2A* and *ADRA2C* promoters in schizophrenia with selective mechanisms in AP-free and AP-treated subjects. To our knowledge, this is the first time that histone PTMs at *ADRA2A* and *ADRA2C* are reported.

Regarding *ADRA2A* epigenetic regulation, both *ADRA2A* promoter-associated H3K4me3 and H3K27me3 were increased in schizophrenia subjects. The relevance of histone methylation processes was already put forward in the GWAS study from the Psychiatric Genomics Consortium [56], whereby histone methylation showed the strongest association with psychiatric disorders. Moreover, H3K27 methyltransferase EZH1 expression has been reported to be increased in PFC of schizophrenia subjects [18]. With our methodology, we cannot assure that regulation of *ADRA2A* promoter by H3K4me3 and H3K27me3 occur at the same cell. Actually, as earlier discussed, *ADRA2A* mRNA expression in brain cortex can be ascribed to GABAergic interneurons, glutamatergic pyramidal neurons and astrocytes [38, 40, 41]. However, levels of H3K4me3 and H3K27me3 at *ADRA2A* promoter showed a positive correlation in human brain (Supplementary Fig. 7) suggesting that these two marks could be concomitantly upregulated in schizophrenia. This parallel increase in H3K4me3 and H3K27me3 marks might be conflicting in the view of their respective permissive and repressive transcriptional influence. Nevertheless, increased H3K4me3 and H3K27me3 at *ADRA2A* promoter region can be explained in the light of bivalent chromatin. Bivalent chromatin refers to those gene promoter regions that show both H3K4me3 and H3K27me3 and thus have characteristics of both active and repressive chromatin [57]. This dual marking keeps genes silent but poised for prompt activation if triggered by certain stimuli. Bivalent chromatin is important in early development [57] but has also been reported in physiological and pathological processes such as aging [58], cancer [59], and autism [60]. Bivalently marked domains are often associated to high-CpG-content promoters [61]. In this sense, estimated CpG island content at *ADRA2A* and *ADRA2C* promoters is 317 and 341 while it is 123 for GAPDH (accessed in UCSC genome browser [62]). Moreover, an annotated list of bivalent chromatin regions in human embrionary stem cells identified *ADRA2A* and *ADRA2C* promoters as bivalent domains based on overlapping H3K4me3 and H3K27me3 peaks [63]. Thus, enriched bivalency at *ADRA2A* promoter depicted by both H3K4me3 and H3K27me3 enhancement in DLPFC of schizophrenia subjects would render *ADRA2A* gene poised for transcriptional action. Upon the right stimulus, this state might be easily activated and lead to increased *ADRA2A* mRNA expression in the cell. This enhanced bivalency would mean that schizophrenia subjects show an epigenetic predisposition for *ADRA2A* mRNA regulation, which might be a specific feature of schizophrenia. Alternatively, as previously discussed, H3K4me3 and H3K27me3 marks at *ADRA2A* promoter might also be due to past effect of antipsychotic treatment. Actually, at *ADRA2A* promoter of AP-treated schizophrenia subjects, H3K4me3 and H3K27me3 were both increased over control values, the difference being statistically significant for H3K27me3.

Another important observation was that in AP-treated schizophrenia subjects, permissive H4K16ac at *ADRA2A* promoter was significantly higher than in matched controls whereas it remained unaltered in AP-free schizophrenia subjects. Increased H4K16ac at *ADRA2A* promoter might be one of the mechanisms tipping the scales in favor of a higher *ADRA2A* mRNA expression in AP-treated schizophrenia subjects. Indeed, among histone H4 PTMs, H4K16ac shows greater individual effect on gene expression than counterparts H4K5, K8, and K12, at least for a subset of genes [64].

Regarding *ADRA2C* epigenetic regulation, present results show that in DLPFC of schizophrenia subjects enhanced repressive H3K27me3, and permissive H3K9ac and H4K5ac coexist at *ADRA2C* promoter region. This pattern of acetylation at histone H3 and H4 together with the repressive methylation at histone H3 suggests that epigenetic modulation of transcriptional activity of *ADRA2C* is also tightly regulated. The histone code depicted by the joint increase in the permissive PTMs H3K9ac and H4K5ac might determine the transcriptional activation of *ADRA2C* gene [65], although precise histone PTM functions still remain unknown [66]. Analysis of schizophrenia subjects based on antipsychotic drug presence revealed the significant upregulation of H3K9ac and H4K5ac in AP-free and AP-treated schizophrenia subjects, respectively. These results may suggest a selective influence of antipsychotic treatment on H4K5ac at *ADRA2C* promoter. However, we acknowledge that the lack of significance in certain subgroups could be due to the human variability of the samples and the reduction of the sample size when dividing the schizophrenia group regarding the presence of antipsychotics in blood.

Modulation of epigenetic mechanisms by antipsychotic treatment is starting to be evaluated. The majority of published articles have evaluated antipsychotic influence on DNA methylation at neurotransmitter-associated candidate genes or genome-wide level [67]. However, data about the effect of antipsychotic drug treatment on histone PTM is scarce. Most relevant findings concern the atypical antipsychotic-induced upregulation of HDAC2, which has been related to histone hypoacetylation at GRM2 promoter [27, 68]. Further evidence on clozapine role in epigenetics is provided by the enhanced expression of HMT mixed-lineage leukemia 1 (*Mll1*) by chronic clozapine [28]. Together with present results, these studies highlight the need for further investigation on antipsychotic drug influence on epigenetic mechanisms.

Studies in postmortem human brain are also complicated by the effect of confounding demographic and methodological factors [69, 70]. According to our results, histone PTMs at *ADRA2A* and *ADRA2C* promoters are influenced by sex, ST, and PMD of the samples. Actually, the effect of sex on epigenetics has been previously described [71] with evidence for HDAC and HMT sex-specific expression [18, 28, 72]. In this sense, our study found that all histone PTMs affected by sex of the subjects showed increased values for females. In any case, the effect of these factors was override by careful matching of schizophrenia and control subjects according to sex, age, PMD, and ST.

In summary, the current study shows *ADRA2A* and *ADRA2C* differential regulation in schizophrenia. The unique upregulation of *ADRA2A* mRNA expression in AP-treated schizophrenia subjects but not in AP-treated rats is likely an outcome of epigenetic predisposition depicted by enhanced bivalent chromatin at *ADRA2A* promoter. On the other hand, increased *ADRA2C* mRNA expression in schizophrenia might be due to the pathophysiology of the disease or to the effect of antipsychotic treatment. Also for *ADRA2C*, histone acetylation and methylation is specifically upregulated at promoter regions. In the light of current and past results [9], the suggested antipsychotic effect potentiation by α_2A_-adrenoceptor agonists or α_2C_-adrenoceptor antagonists deserves further study [5]. A final warning due to the specificity of histone PTMs at different genes is that the impact of coadjuvant antipsychotic and HDAC inhibitors [31] at relevant gene clusters should also be carefully studied.

## Supplementary information

Supplementary data
