# Supplementary material for: Differential brain ADRA2A and ADRA2C gene expression and epigenetic regulation in schizophrenia. Effect of antipsychotic drug treatment
Source: Transl Psychiatry. 2021 Dec 20;11:643. doi: 10.1038/s41398-021-01762-4 (PMC8688495; doi:10.1038/s41398-021-01762-4)
Supplement: Supplementary file 1 — Supplementary data [file 41398_2021_1762_MOESM1_ESM.docx]

**Supplementary data**

Table 1. Demographic and toxicological characteristics of schizophrenia and control subjects included in the mRNA expression study.

| Case | Sex (M/F) | Age (years) | PMD (hours) | ST (months) | RIN | Cause of death | | Antipsychotic drugs in blood | | Other drugs in blood |
| --- | --- | --- | --- | --- | --- | --- | --- | --- | --- | --- |
| Antipsychotic free S subjects (n=10) and matched controls (n=10) | | | | | | | | | | |
| S1 | M | 48 | 20 | 141 | 7.80 | Suicidal (train) | |  | |  |
| C1 | M | 47 | 17 | 162 | 7.90 | Natural (CRF) | |  | | Nordiazepam |
| S2 | M | 45 | 3 | 125 | 6.70 | Suicidal (jumping) | |  | | Nordiazepam |
| C2 | M | 48 | 7 | 162 | 7.10 | Accidental (traffic) | |  | |  |
| S3 | F | 37 | 58 | 109 | 7.20 | Suicidal (drug intoxication) | |  | | Heroin  Diazepam |
| C3 | F | 36 | 38 | 233 | 8.20 | Homicide | |  | | Diazepam  Heroin  Cocaine |
| S4 | M | 46 | 22 | 100 | 6.00 | Suicidal (jumping) | |  | |  |
| C4 | M | 41 | 14 | 80 | 9.10 | Natural  (heart attack) | |  | |  |
| S5 | F | 37 | 26 | 68 | 7.40 | Suicidal (jumping) | |  | | Lorazepam |
| C5 | F | 38 | 22 | 65 | 6.80 | Accidental  (run over) | |  | |  |
| S6 | M | 48 | 11 | 69 | 7.00 | Suicidal (jumping) | |  | |  |
| C6 | M | 49 | 8 | 62 | 8.70 | Natural (CRF) | |  | |  |
| S7 | M | 35 | 5 | 71 | 8.50 | Suicidal (hanging) | |  | |  |
| C7 | M | 37 | 6 | 254 | 7.30 | Accidental (traffic) | |  | | Ethanol |
| S8 | M | 45 | 18 | 78 | 6.70 | Suicidal (jumping) | |  | |  |
| C8 | M | 47 | 15 | 63 | 7.70 | Accidental (traffic) | |  | |  |
| S9 | M | 34 | 15 | 81 | 6.70 | Natural (CRF) | |  | |  |
| C9 | M | 34 | 17 | 40 | 8.00 | Accidental (traffic) | |  | |  |
| S10 | M | 52 | 7 | 85 | 6.60 | Suicidal (jumping) | |  | | Lormetazepam |
| C10 | M | 51 | 13 | 65 | 5.90 | Accidental (traffic) | |  | | Ethanol |
| Antipsychotic free S subjects (n=10) and matched controls (n=10) | | | | | | | | | | |
| Schizophrenia | 8M/2F | 43±2 | 19±5 | 93±8 | 7.06±0.71 |  | |  | |  |
| Controls | 8M/2F | 43±2 | 16±3 | 119±25 | 7.67±0.94 |  | |  | |  |
| Antipsychotic treated S subjects (n=9) and matched controls (n=9) | | | | | | | | | | |
| S11 | M | 44 | 7 | 150 | 8.30 | Natural (CRF) | | Clotiapine  Levomepromazine | | Nordiazepam |
| C11 | M | 44 | 23 | 69 | 8.30 | Accidental  (run over) | |  | |  |
| S12 | M | 35 | 11 | 89 | 8.00 | Natural (CRF) | | Clozapine | | Clomipramine  Nordiazepam |
| C12 | M | 36 | 18 | 159 | 7.30 | Accidental (work) | |  | | Ethanol |
| S13 | F | 60 | 23 | 73 | 6.00 | Natural  (peritonitis) | | Clozapine  Amisulpride | | Lorazepam |
| C13 | F | 60 | 48 | 79 | 7.80 | Natural (cerebral haemorrhage) |  | |  | |
| S14 | M | 56 | 12 | 78 | 6.70 | Natural (CRF) | | Olanzapine  Clotiapine | |  |
| C14 | M | 54 | 16 | 69 | 8.70 | Accidental (work) | |  | | Ethanol |
| S15 | M | 41 | 17 | 44 | 5.60 | Homicide  (white weapon) | | Clozapine | | Alprazolam |
| C15 | M | 40 | 18 | 158 | 7.60 | Accidental (work) | |  | | Ethanol |
| S16 | M | 42 | 14 | 89 | 5.90 | Suicidal  (toxic ingestion) | | Risperidone | | Nordiazepam |
| C16 | M | 43 | 20 | 61 | 7.40 | Natural (CRF) | |  | |  |
| S17 | M | 36 | 8 | 110 | 9.40 | Suicidal  (toxic ingestion) | | Olanzapine | |  |
| C17 | M | 32 | 4 | 42 | 8.50 | Accidental  (run over) | |  | |  |
| S18 | F | 48 | 17 | 127 | 6.80 | Suicidal (jumping) | | Olanzapine  Clotiapine | |  |
| C18 | F | 50 | 11 | 86 | 8.00 | Natural (CRF) | |  | |  |
| S19 | F | 42 | 14 | 136 | 6.90 | Suicidal  (toxic ingestion) | | Clozapine  Sulpiride  Quetiapine | | Nordiazepam |
| C19 | F | 41 | 22 | 58 | 6.90 | Accidental (crushed) | |  | |  |
| Antipsychotic treated S subjects (n=9) and matched controls (n=9) | | | | | | | | | | |
| Schizophrenia | 6M/3F | 45±3 | 14±2 | 100±11 | 7.07±1.26 |  | |  | |  |
| Controls | 6M/3F | 44±3 | 20±4 | 87±14 | 7.83±0.6 |  | |  | |  |
| All S subjects (n=19) and matched controls (n=19) | | | | | | | | | | |
| Schizophrenia | 14M/5F | 44±2 | 16±3 | 96±7 | 7.06±0.98 |  | |  | |  |
| Controls | 14M/5F | 44±2 | 18±2 | 104±15 | 7.75±0.78 |  | |  | |  |

Group values are means ± S.E.M. M, male; F, female; C, control subjects; S, schizophrenia subjects; PMD, postmortem delay; ST, storage time; CRF, cardiorespiratory failure.

Schizophrenia cases and controls have been previously used in the evaluation of α_2A_- and α_2C_-adrenoceptor expression in different subcellular fractions [Brocos-Mosquera et al. *European Neuropsychopharmacology* **52**, 3-11 (2021)].

Table 2. Demographic and toxicological characteristics of schizophrenia and control subjects included in the epigenetic study.

| Case | Sex (M/F) | Age (years) | PMD  (hours) | ST  (months) | Cause of death | Antipsychotic drugs in blood | Other drugs  in blood |
| --- | --- | --- | --- | --- | --- | --- | --- |
| Antipsychotic free S subjects (n=12) and matched controls (n=12) | | | | | | | |
| S1 | M | 31 | 14 | 145 | Suicidal (jumping) |  | Lorazepam |
| C1 | M | 32 | 4 | 42 | Accidental  (run over) |  |  |
| S2 | M | 48 | 20 | 141 | Suicidal (train) |  |  |
| C2 | M | 47 | 17 | 162 | Natural (CRF) |  | Nordiazepam |
| S3 | M | 45 | 3 | 125 | Suicidal (jumping) |  | Nordiazepam |
| C3 | M | 48 | 7 | 162 | Accidental (traffic) |  |  |
| S4 | F | 37 | 58 | 109 | Suicidal (drug intoxication) |  | Heroin, Diazepam |
| C4 | F | 36 | 38 | 233 | Homicide |  | Diazepam, Heroin, Cocaine |
| S5 | M | 46 | 22 | 100 | Suicidal (jumping) |  |  |
| C5 | M | 46 | 24 | 86 | Natural  (heart attack) |  |  |
| S6 | F | 37 | 26 | 68 | Suicidal (jumping) |  | Lorazepam |
| C6 | F | 38 | 22 | 65 | Accidental  (run over) |  |  |
| S7 | M | 48 | 11 | 69 | Suicidal (jumping) |  |  |
| C7 | M | 49 | 8 | 62 | Natural (CRF) |  |  |
| S8 | M | 35 | 5 | 71 | Suicidal (hanging) |  |  |
| C8 | M | 37 | 6 | 254 | Accidental (traffic) |  | Ethanol |
| S9 | F | 59 | 9 | 76 | Natural (CRF) |  |  |
| C9 | F | 57 | 4 | 114 | Accidental (traffic) |  |  |
| S10 | M | 45 | 18 | 78 | Suicidal (jumping) |  |  |
| C10 | M | 47 | 15 | 63 | Accidental (traffic) |  |  |
| S11 | M | 34 | 15 | 81 | Natural (CRF) |  |  |
| C11 | M | 34 | 17 | 40 | Accidental (traffic) |  |  |
| S12 | M | 52 | 7 | 85 | Suicidal (jumping) |  | Lormetazepam |
| C12 | M | 51 | 13 | 65 | Accidental (traffic) |  | Ethanol |
| Antipsychotic free S subjects (n=12) and matched controls (n=12) | | | | | | | |
| Schizophrenia | 9M/3F | 43±2 | 17±4 | 96±8 |  |  |  |
| Controls | 9M/3F | 44±2 | 15±3 | 112±21 |  |  |  |
| Antipsychotic treated S subjects (n=12) and matched controls (n=12) | | | | | | | |
| S13 | M | 44 | 7 | 150 | Natural (CRF) | Clotiapine, Levomepromazine | Nordiazepam |
| C13 | M | 44 | 23 | 69 | Accidental  (run over) |  |  |
| S14 | F | 30 | 28 | 133 | Suicidal (drug intoxication) | Haloperidol | Lorazepam |
| C14 | F | 30 | 18 | 76 | Accidental (traffic) |  |  |
| S15 | M | 35 | 11 | 89 | Natural (CRF) | Clozapine | Clomipramine, Nordiazepam |
| C15 | M | 36 | 18 | 159 | Accidental (work) |  | Ethanol |
| S16 | F | 60 | 23 | 73 | Natural  (peritonitis) | Clozapine  Amisulpride | Lorazepam |
| C16 | F | 60 | 48 | 79 | Natural (cerebral haemorrhage) |  |  |
| S17 | M | 56 | 12 | 78 | Natural (CRF) | Olanzapine Clotiapine |  |
| C17 | M | 54 | 16 | 69 | Accidental (work) |  | Ethanol |
| S18 | M | 41 | 17 | 44 | Homicide  (white weapon) | Clozapine | Alprazolam |
| C18 | M | 40 | 18 | 158 | Accidental (work) |  | Ethanol |
| S19 | M | 41 | 11 | 45 | Suicidal  (white weapon) | Clotiapine, Quetiapine | Lorazepam |
| C19 | M | 41 | 14 | 80 | Natural  (heart attack) |  |  |
| S20 | M | 42 | 14 | 89 | Suicidal  (toxic ingestion) | Risperidone | Nordiazepam |
| C20 | M | 43 | 20 | 61 | Natural (CRF) |  |  |
| S21 | M | 36 | 8 | 110 | Suicidal  (toxic ingestion) | Olanzapine |  |
| C21 | M | 36 | 23 | 70 | Accidental (crushed) |  |  |
| S22 | F | 28 | 22 | 125 | Suicidal (jumping) | Risperidone |  |
| C22 | F | 32 | 19 | 140 | Accidental (traffic) |  | Ethanol |
| S23 | F | 48 | 17 | 127 | Suicidal (jumping) | Olanzapine, Clotiapine |  |
| C23 | F | 50 | 11 | 86 | Natural (CRF) |  |  |
| S24 | F | 42 | 14 | 136 | Suicidal  (toxic ingestion) | Clozapine,  Sulpiride, Quetiapine | Nordiazepam |
| C24 | F | 41 | 22 | 58 | Accidental (crushed) |  |  |
| Antipsychotic treated S subjects (n=12) and matched controls (n=12) | | | | | | | |
| Schizophrenia | 7M/5F | 42±3 | 15±2 | 100±10 |  |  |  |
| Controls | 7M/5F | 42±3 | 21±3 | 92±11 |  |  |  |
| All S subjects (n=24) and matched controls (n=24) | | | | | | | |
| Schizophrenia | 16M/8F | 43±2 | 16±2 | 98±6 |  |  |  |
| Controls | 16M/8F | 43±2 | 18±2 | 102±12 |  |  |  |

Group values are means ± S.E.M. M, male; F, female; C, control subjects; S, schizophrenia subjects; PMD, postmortem delay; ST, storage time; CRF, cardiorespiratory failure.

Schizophrenia cases and controls have been previously used in the evaluation of α_2A_- and α_2C_-adrenoceptor expression in different subcellular fractions [Brocos-Mosquera et al. *European Neuropsychopharmacology* **52**, 3-11 (2021)].

Table 3. Sex, age, PMD and ST of those schizophrenia subjects that were suicide victims. Quantitative variables are expressed as mean ± SEM (standard error of the mean).

|  | **ALL** | **AP-free** | **AP-treated** |
| --- | --- | --- | --- |
|  | **17 S** | **7 S** | **10 S** |
| **Sex (M/F)** | 11M/6F | 3M/4F | 8M/2F |
| **Age (years)** | 41±2 | 42±2 | 38±3 |
| **PMD (hours)** | 18±3 | 18±5 | 16±3 |
| **ST (months)** | 103±7 | 99±9 | 109±12 |

M, male; F, female; S, schizophrenia subjects; AP-free, antipsychotic-free; AP-treated, antipsychotic-treated; PMD, postmortem delay; ST, storage time.

Table 4. Demographic and toxicological characteristics of suicide victims with major depression and matched controls included in the mRNA expression study.

| Case | | Sex (M/F) | | Age (years) | | | PMD (hours) | | ST (months) | | RIN | | Cause of death | Antidepressant drugs in blood | Other drugs in blood |
| --- | --- | --- | --- | --- | --- | --- | --- | --- | --- | --- | --- | --- | --- | --- | --- |
| MD1 | | F | | 35 | | | 23 | | 168 | | 5.2 | | Suicidal (jumping) |  |  |
| C1 | | F | | 35 | | | 22 | | 204 | | 7.8 | | Accidental (traffic) |  |  |
| MD2 | | F | | 73 | | | 17 | | 48 | | 6.6 | | Suicidal (white weapon) |  |  |
| C2 | | F | | 74 | | | 19 | | 60 | | 4.6 | | Accidental (traffic) |  |  |
| MD3 | | F | | 79 | | | 13 | | 36 | | 7.6 | | Suicidal (jumping) |  |  |
| C3 | | F | | 77 | | | 13 | | 108 | | 7.6 | | Accidental (run over) |  |  |
| MD4 | | M | | 78 | | | 22 | | 60 | | 7.5 | | Suicidal (hanging) |  |  |
| C4 | | M | | 78 | | | 22 | | 168 | | 7.4 | | Accidental (run over) |  |  |
| MD5 | | F | | 56 | | | 24 | | 156 | | 5.9 | | Suicidal (jumping) |  |  |
| C5 | | F | | 57 | | | 4 | | 36 | | 7.2 | | Accidental (traffic) |  |  |
| MD6 | | F | | 50 | | | 19 | | 72 | | 7.5 | | Suicidal (jumping) | Venlafaxine, mirtazapine |  |
| C6 | | F | | 50 | | | 31 | | 132 | | 5.9 | | Accidental (traffic) |  |  |
| MD7 | | F | | 35 | | | 25 | | 60 | | 6.5 | | Suicidal (jumping) | Venlafaxine, mirtazapine | Benzodiazepines, ∆^9^tetrahydrocannabinol (THC) |
| C7 | | F | | 35 | | | 24 | | 120 | | 7.1 | | Accidental (traffic) |  |  |
| MD8 | | M | | 33 | | | 14 | | 60 | | 8 | | Suicidal (jumping) | Venlafaxine | Benzodiazepines |
| C8 | | M | | 31 | | | 13 | | 48 | | 8.1 | | Accidental (traffic) |  | Ethanol |
| MD9 | | M | | 43 | | | 34 | | 36 | | 5.8 | | Suicidal (hanging) | Citalopram | Benzodiazepines |
| C9 | | M | | 43 | | | 15 | | 180 | | 8.2 | | Accidental (traffic) |  |  |
| MD10 | | F | | 49 | | | 18 | | 24 | | 4.4 | | Suicidal (drowning) | Fluoxetine |  |
| C10 | | F | | 58 | | | 20 | | 120 | | 7.9 | | Natural (heart failure) |  |  |
| MD11 | | M | | 50 | | | 9 | | 72 | | 7.2 | | Suicidal (drug intoxication) | Venlafaxine | Quetiapine |
| C11 | M | | | | 48 | 7 | | 84 | | | | 7.2 | Accidental (traffic) |  |  |
| MD12 | | F | | 35 | | | 7 | | 24 | | 5.6 | | Suicidal (jumping) | Duloxetine, fluoxetine | Cocaine |
| C12 | | F | | 36 | | | 8 | | 60 | | 6.8 | | Natural (heart failure) |  |  |
| MD13 | | | M | | 75 | 17 | | | 24 | 8.3 | | | Suicidal (jumping) | Venlafaxine, mirtazapine | Benzodiazepines |
| C13 | | M | | 76 | | | 24 | | 24 | | 7.8 | | Accidental (traffic) |  | Ethanol |
| All MD subjects (n=13) and matched controls (n=13) | | | | | | | | | | | | | | | |
| Depression | | 5M/8F | | 53±5 | | | 19±2 | | 65±13 | | 6.62±0.33 | |  |  |  |
| Controls | | 5M/8F | | 54±5 | | | 17±2 | | 103±16 | | 7.2±0.28 | |  |  |  |

Group values are means ± S.E.M. M, male; F, female; C, control subjects; MD, depression subjects; PMD, postmortem delay; ST, storage time.

Depression cases and controls have been previously used in the evaluation of 5-HT_2A_ and mGlu2/3 receptors [Muguruza et al. *Neuropharmacology* **86**, 311-318 (2014)].

Table 5. Description and concentration of primary antibodies.

| Primary antibodies | | | | |
| --- | --- | --- | --- | --- |
| Target | **Description** | **Manufacturer** | **Catalog #** | **Ab dilution** |
| H3K4me3 | Polyclonal rabbit anti-trimethyl-histone H3 on lysine 4 | Merck Millipore | 07-473 | 1:333 |
| H3K27me3 | Polyclonal rabbit anti-trimethyl-histone H3 on lysine 27 | Merck Millipore | 07-449 | 1:200 |
| AcH3 | Polyclonal rabbit anti-acetyl-histone H3 | Merck Millipore | 06-559 | 1:200 |
| AcH3K9 | Polyclonal rabbit anti-acetyl-histone H3 on lysine 9 | Merck Millipore | 07-352 | 1:200 |
| AcH3K27 | Polyclonal rabbit anti-acetyl-histone H3 on lysine 27 | Merck Millipore | 07-360 | 1:167 |
| AcH4K5 | Polyclonal rabbit anti-acetyl-histone H4 on lysine 5 | Merck Millipore | 07-327 | 1:100 |
| AcH4K16 | Polyclonal rabbit anti-acetyl-histone H4 on lysine 16 | Merck Millipore | 07-329 | 1:200 |
| IgG | Polyclonal rabbit-anti-IgG | Merck Millipore | 12-370 | 1:333 |

Table 6. PCR primer pairs for ChIP and qPCR assay in postmortem human brain samples and rat brain.

| Sample | Sample amount (ng) | Target gene | Type of primer | Sequence | Primer concentration (µM) | Assay  buffer | Reaction efficiency (%) | Amplifi-cation product  (bp) |
| --- | --- | --- | --- | --- | --- | --- | --- | --- |
| Human cDNA | 20 | ***ADRA2A*** | Forward | 5’ -GAGCAAGCACTGGACTACAA-3’ | 0.8 | Power Sybr Green | 99 | 99 |
|  |  |  | Reverse | 5’-AGAGAGAGAGTTGGGAATGGA-3’ | 0.8 |  |  |  |
|  | 20 | ***ADRA2C*** | Forward | 5’-TTCACCTTTGTGCTGGCT-3’ | 0.8 | Power Sybr Green | 91 | 75 |
|  |  |  | Reverse | 5’- CAGGCTGTAGCTGAAGAAGAA-3’ | 0.8 |  |  |  |
| Rat cDNA | 4 | ***Adra2a*** | Forward | 5’-TCCTGAGAGGGAAGGGATTT-3’ | 0.4 | Fast Sybr Green | 110 | 70 |
|  |  |  | Reverse | 5’-GTCTTGGTCCATCGCTGTAATA-3’ | 0.4 |  |  |  |
|  | 4 | ***Adra2c*** | Forward | 5’-CTCTGGCTGCCTGGACTT-3’ | 0.4 | Fast Sybr Green | 101 | 60 |
|  |  |  | Reverse | 5’-GTTGGTCCCCCTATGTACCC-3’ | 0.4 |  |  |  |
| Human genomic DNA |  | ***ADRA2A*** | Forward | 5’-TAACTCACACCGGAGGTTACT-3’ | 0.1 | Fast Sybr Green | 107 | 125 |
|  |  |  | Reverse | 5’-ACGGAGCAGAACGCAAC-3’ | 0.1 |  |  |  |
|  |  | ***ADRA2C*** | Forward | 5’-TGGTGACTTTGAACACCTCTC-3’ | 0.2 | Fast Sybr Green | 102 | 150 |
|  |  |  | Reverse | 5’-TGCTGGAGCATGAATCATAACTA-3’ | 0.2 |  |  |  |
|  |  | ***GAPDH*** | Forward | 5’-TACTAGCGGTTTTACGGGCG-3’ | 0.1 | Fast Sybr Green | 108 | 165 |
|  |  |  | Reverse | 5’-TCGAACAGGAGGAGCAGAGAGCGA-3’ | 0.1 |  |  |  |

Table 7. Taqman assays used in qPCR experiments in postmortem human brain and in rat brain.

| Sample | Gene | Gene name | Taqman assay ID |
| --- | --- | --- | --- |
| Human cDNA | *GAPDH* | Glyceraldehyde 3-phosphate dehydrogenase | 43333762F |
|  | *RPS13* | Ribosomal protein S13 | Hs01945436_u1 |
| Rat cDNA | *Gapdh* | Glyceraldehyde 3-phosphate dehydrogenase | 4352932E |
|  | *Rps29* | Ribosomal protein S29 | Mm02342448_gH |


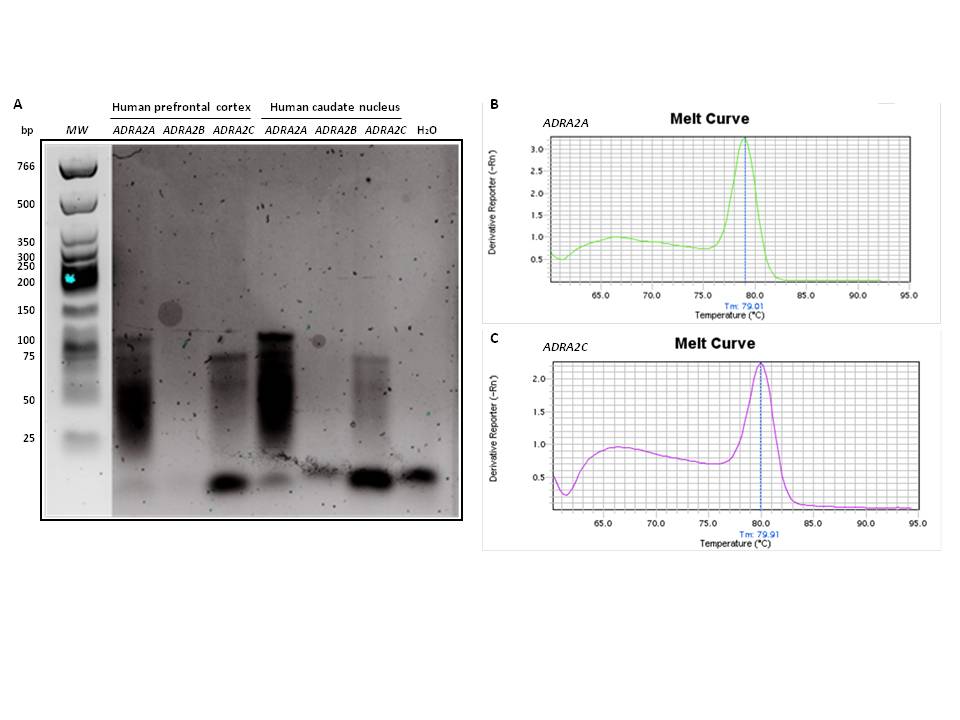


Figure 1. Characterization of qPCR assay for quantification of *ADRA2A* and *ADRA2C* mRNA expression in postmortem human brain. A. Amplification products of *ADRA2A* and *ADRA2C* mRNA in human prefrontal cortex and human caudate nucleus were electrophoretically separated for correct size verification. Molecular grade H_2_O was run as negative control. B. Melting curve of qPCR amplification product obtained in *ADRA2A* mRNA expression study. C. Melting curve of qPCR amplification product obtained in *ADRA2C* mRNA expression study. These analyses confirmed the presence of a main peak in all cases.

Figure 2. Characterization of qPCR assays for specific *ADRA2A*, *ADRA2C* and *GAPDH* genomic DNA amplification on immunoprecipitated chromatin. Amplification products were electrophoretically separated for correct size verification. Melting curves of qPCR amplification products confirmed the presence of a main peak in all cases.


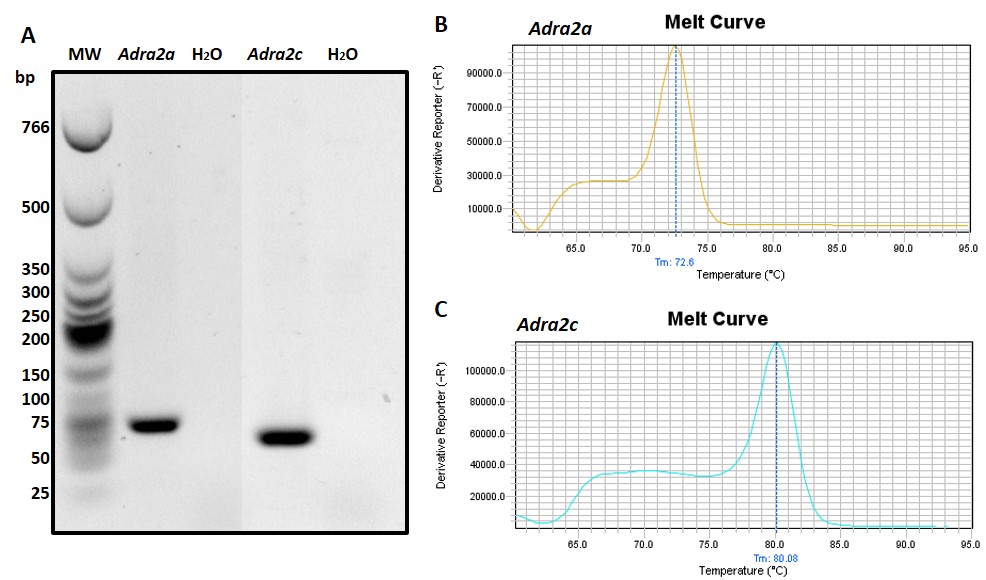


Figure 3. Characterization of qPCR for quantification of *Adra2a* and *Adra2c* mRNA expression in rat brain cortex. A. Amplification products of *Adra2a* and *Adra2c* mRNA in rat brain cortex were electrophoretically separated for correct size verification. Molecular grade H_2_O was run as negative control. B. Melting curve of qPCR amplification product obtained in *Adra2a* mRNA expression study. C. Melting curve of qPCR amplification product obtained in *Adra2c* mRNA expression study. These analyses confirmed the presence of a main peak in all cases.


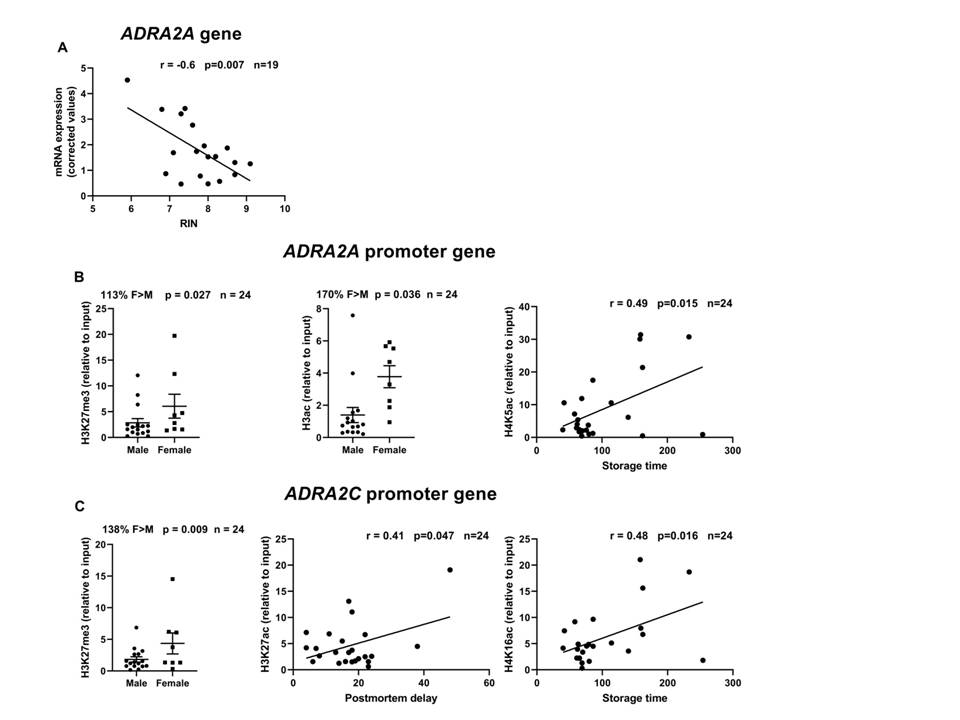


Figure 4. A. Effect of RIN on corrected *ADRA2A* mRNA expression in DLPFC of control subjects studied by linear regression analyses, where r values are the Pearson’s correlation coefficients and the lines represent the regression of the correlation (for RIN and *ADRA2A* mRNA expression: y=8.734-0.895x, n=19). B. Effect of sex, postmortem delay and storage time in postmortem human brain histone posttranslational modifications (PTMs) at *ADRA2A* and *ADRA2C* promoter regions. Histone PTMs at *ADRA2A* and *ADRA2C* promoters in males and females was compared by two-tailed unpaired Student’s t-test. The effect of storage time and postmortem delay was studied by linear regression analyses where r values are the Pearson’s correlation coefficients and the lines represent the regressions of the correlations (for storage time and H4K5ac at *ADRA2A* promoter gene: y=0.011+0.085x, n=24; for PMD and H3K27ac at *ADRA2C* promoter gene: y=1.495+0.1795x, n=24; for storage time and H4K16ac at *ADRA2C* promoter gene: y=1.52+0.045x, n=24). Statistical significance is denoted by *p* < 0.05.





Figure 5. Corrected *ADRA2A* and *ADRA2C* mRNA expression in DLPFC of suicide victims with major depression (MD, n=12) and their respective matched controls (C). Data are shown as mean ± SEM.


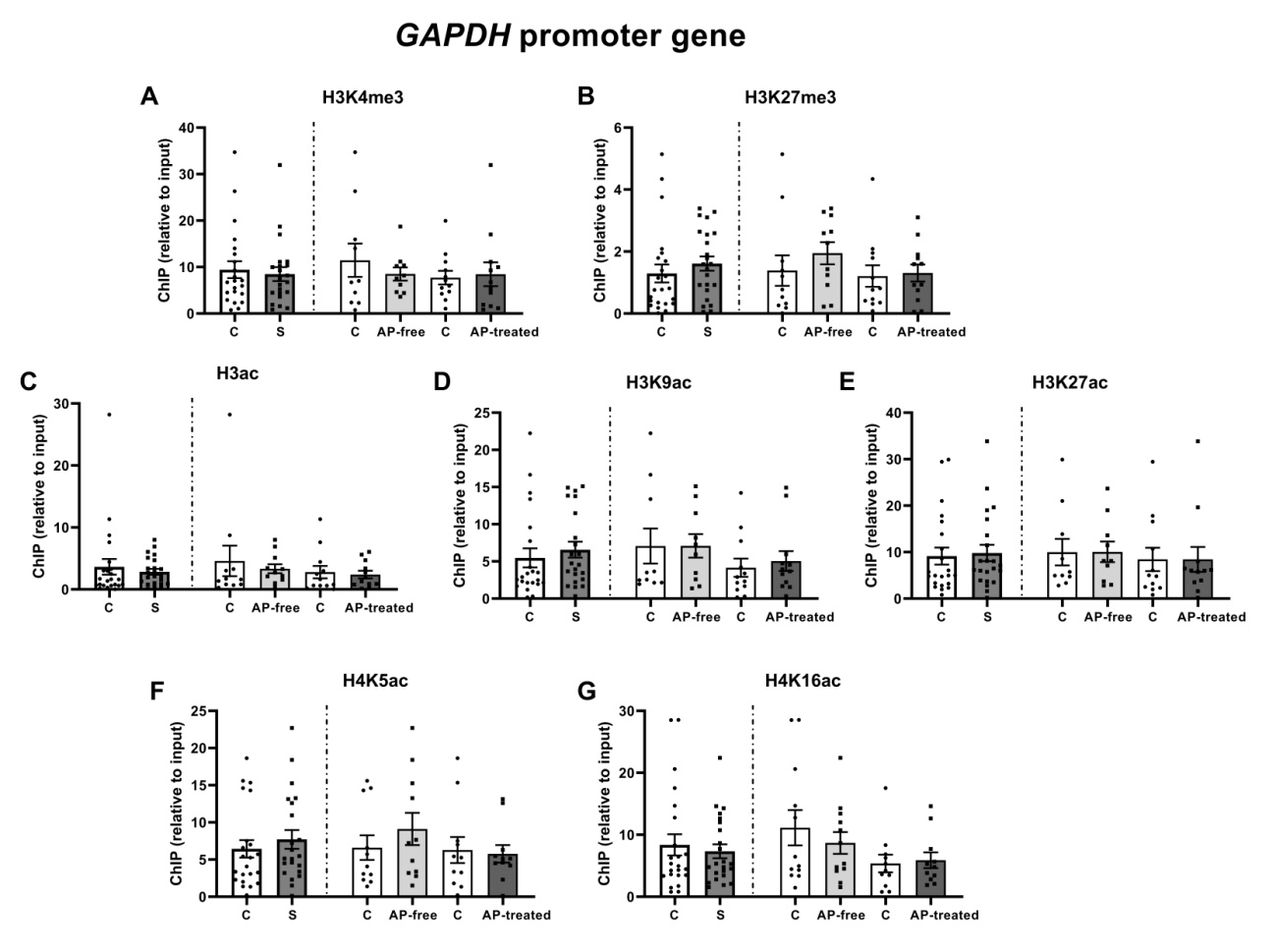


Figure 6. Histone 3 methylation (H3K4me3 and H3K27me3) and acetylation (H3ac, H3K9ac, H3K27ac) and histone 4 acetylation (H4K5ac and H4K16ac) at promoter region of *GAPDH* gene. Data are shown as mean ± SEM.


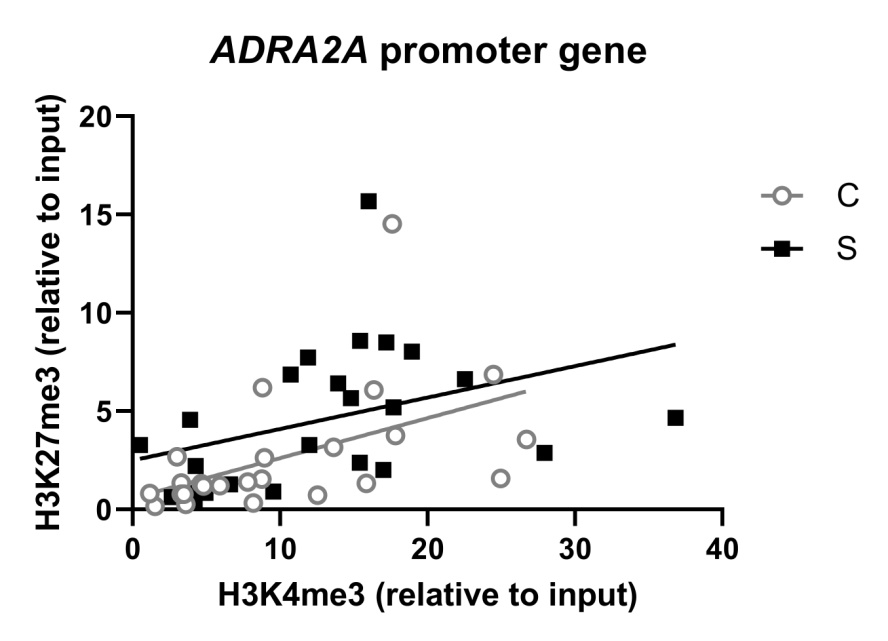


Figure 7. Correlation between H3K27me3 and H3K4me3 at *ADRA2A* promoter gene in DLPFC of controls (C, white circles) and subjects with schizophrenia (S, black squares) studied by linear regression analyses, where r values are the Pearson’s correlation coefficients and the lines represent the regression of the correlation (for control subjects: y=0.577+0.203x,r=0.498 *p*=0.013, n=24; for schizophrenia subjects: y=2.491+0.160x, r=0.38, *p*=0.067, n=24).
